# Current Flow in Nerves and Mitochondria: An Electro-Osmotic Approach

**DOI:** 10.3390/biom15081063

**Published:** 2025-07-22

**Authors:** Robert S. Eisenberg

**Affiliations:** 1 Department of Applied Mathematics, Illinois Institute of Technology, Chicago, IL 60616, USA; bob.eisenberg@gmail.com; 2 Department of Physiology and Biophysics, Rush University Medical Center, Chicago, IL 60612, USA; 3 Department of Biomedical Engineering, University of Illinois, Chicago, IL 60607, USA

**Keywords:** mitochondria, electro-osmotic, action potential, Kirchhoff current law, chemiosmotic

## Abstract

The electrodynamics of current provide much of our technology, from telegraphs to the wired infrastructure powering the circuits of our electronic technology. Current flow is analyzed by its own rules that involve the Maxwell Ampere law and magnetism. Electrostatics does not involve magnetism, and so current flow and electrodynamics cannot be derived from electrostatics. Practical considerations also prevent current flow from being analyzed one charge at a time. There are too many charges, and far too many interactions to allow computation. Current flow is essential in biology. Currents are carried by electrons in mitochondria in an electron transport chain. Currents are carried by ions in nerve and muscle cells. Currents everywhere follow the rules of current flow: Kirchhoff’s current law and its generalizations. The importance of electron and proton flows in generating ATP was discovered long ago but they were not analyzed as electrical currents. The flow of protons and transport of electrons form circuits that must be analyzed by Kirchhoff’s law. A chemiosmotic theory that ignores the laws of current flow is incorrect physics. Circuit analysis is easily applied to short systems like mitochondria that have just one internal electrical potential in the form of the Hodgkin Huxley Katz (HHK) equation. The HHK equation combined with classical descriptions of chemical reactions forms a computable model of cytochrome c oxidase, part of the electron transport chain. The proton motive force is included as just one of the components of the total electrochemical potential. Circuit analysis includes its role just as it includes the role of any other ionic current. Current laws are now needed to analyze the flow of electrons and protons, as they generate ATP in mitochondria and chloroplasts. Chemiosmotic theory must be replaced by an electro-osmotic theory of ATP production that conforms to the Maxwell Ampere equation of electrodynamics while including proton movement and the proton motive force.

## 1. Author Statement

I am most grateful to the anonymous authors and artists who made the figures reproduced here. I have made zero intellectual contribution to the information in the figures beyond Ref. [1].

The figures convincingly document the importance of electron and proton flow as seen in the experimental literature of the respiratory chain, independent of theoretical discussion using current laws.

The figures in this paper are taken from public domain websites. It is not useful to provide individual references to these websites because they change so often. The websites all allow copying, as best I can tell. If I have inadvertently failed to give proper attribution, I will make corrections and of course apologize.

I repeat I have made no intellectual contribution to the figures or the work they report demonstrating the movement of charges. My original contribution may have been to point out that the movement of charges is an electrical current subject to the Maxwell Ampere equation of electrodynamics. The treatment of charge movement includes the flux of protons and also the proton motive force that helps drive the flux. The treatment of charge movement includes the movement of all charge carriers and their driving forces. It includes the the chemical and electrical gradients of potential, concentrations, and voltages.

## 2. Attribution and Sources

Current flow follows its own rules set by the physics of magnetism as well as electricity—the Maxwell equations of electrodynamics [2,3,4] including the Maxwell Ampere law. Electrostatics does not involve magnetism. Current flow and electrodynamics cannot be derived from electrostatics because electrostatics does not involve magnetism. Indeed, current only appears in one Maxwell equation, the Maxwell Ampere law that defines the source of (and the curl of) the magnetic field [5,6,7].

The rules of current flow apply to all currents, currents carried by ions, protons, or electrons in all systems, physical and biological, experimental, or theoretical including simulations. Mitochondria are not an exception. The chemiosmotic hypothesis [8,9,10,11,12] is not an exception. The electrodynamic rules of current flow include the proton motive force along with the driving forces and flows of any charge carrier.

The original Maxwell equations are constitutive equations of materials that depend on a crude dielectric approximation to the polarization properties of matter [13]. For solids, see Reference [5], Section 4.2.3. For liquids and ionic solutions, see [14]. The universal nature of the Maxwell equations emerges when they are written without the dielectric approximation so they are no longer constitutive equations. In fact, in that formulation the Maxwell equations do not depend explicitly on material parameters.

Polarization describes the movement and compressibility of charged materials when electrical forces are applied. The range of possible motions is enormous, particularly those of ionic solutions and proteins [13,15,16] and includes both (electro) diffusion and compression of charge density. Almost all of life occurs in ionic solutions and depends on proteins. The original Maxwell equations, with their oversimplified model of polarization, cannot possibly describe ionic solutions and proteins, using just one dielectric constant (a single real positive number). The properties are far too diverse, varying with time (frequency), ionic concentration, ionic composition, pH, and more or less any variable that applies to the liquid phase. The universal nature of the Maxwell equations is not apparent when the restrictive dielectric approximation is used. The importance (and role) of the Maxwell equations has not always been apparent in biological systems perhaps because those equations have usually included a dielectric approximation inappropriate for proteins and biological systems.

The universal nature of the Maxwell equations is seen more clearly once the dielectric approximation is removed and replaced by models of polarization specific for the system of interest [13,15,16,17,18]. The dielectric constant should be taken as a (single positive real) constant only when experimental estimates or theoretical models are not available, in my view. That is often the case when teaching the Maxwell equations, or when models are constructed of new situations. But one must always remember that using the Maxwell equations with a single dielectric constant obscures their universal nature. Polarization can be analyzed in an energetic analysis by the variational treatment of complex fluids that specifies the free energy of polarization [19]: Section 3.6.

It should be clearly understood that the rules of current flow are true on all time scales that have been studied. Total current flow forms a very special physical field. It has no sources or sinks in the ordinary sense of those words. The current flow is without divergence, called solenoidal in the physics and mathematics literature [20], particularly p. 29. It comes from dipolar sources, not from single charges or their layers. These properties are implied by the Maxwell equations of electrodynamics without approximation and ***without any adjustable parameters***. These properties are as universal and as exact as any physics that is known.

The Maxwell equations certainly apply to mitochondria as they do to nerve fibers where they have been used for some eighty years. They certainly include the movement of protons and the proton motive forces that help drive them. Chemiosmotic theories must be a subset of electrodynamics, if electrodynamics is universal.

Total current (as discussed later) in two-dimensional systems like circuits flows in loops on all time scales. At any instant in time, the total current anywhere in an isolated loop (like a single-series circuit) is exactly the same even though the physical nature of the current is very different in different places within the loop, i.e., within the single series circuit (see [21] for a detailed physical explanation). As Landauer put it [22] on p. 112, “It is, after all, the sum of electron current and displacement current which has no divergence. One of those two components can take over from the other.”

The behavior of the total current is not intuitive. Intuition usually considers fluxes that can be temporarily stored before they eventually “balance out”. Intuition usually considers the flux of specific species, not the flow of total current. ***Intuition ignores the fact that current appears in ONLY ONE of the Maxwell equations*** and that describes the source of magnetism. The simple fact is that the properties of current flow are not derivable from electrostatics because electrostatics does not include magnetism or the flux and displacement current that are the source of the magnetic field. Electrodynamics cannot be derived from electrostatics, which is exactly why Maxwell had to revise the original statement of Ampere’s law [5].

The flux of each species is NOT the same in every circuit element in a series circuit [21]. The total current has very different properties. The total current is exactly the same at all times in every element in the series circuit, with no exceptions. Total current is never stored in any time interval however brief. It is never stored. Ever.

The special properties of total current are seen strikingly in series circuits, i.e., in the individual branches of complex circuits. Series circuits including chemical reactions are very important in biology. If one of the circuit elements is a classical series chemical reaction described by the law of mass action, the total current ***at every instant and in every step of the reaction*** is the same no matter what the speed or rate constant of the reaction. This property provides an important constraint that requires analysis to include the displacement current in the steps of the chemical reaction (that is proportional to the time derivative of the electric field). The displacement current is discussed in more detail later in this paper. Few chemical reactions include the displacement current in their traditional description or analysis. In particular, the chemiosmotic theory does not mention displacement current despite the large capacitance of the mitochondrial membrane and the speed of chemical reactions involving ATP synthase (among others). Of course, the chemiosmotic theory does not mention electrical current at all, as far as I can tell, or the Kirchhoff law (and its generalizations) that governs current flow (Appendix B). In contrast, an electro-osmotic theory includes the proton movement and proton motive force of the chemiosmotic theory as a component of the total system, as it includes the movement and driving force of any ion.

The properties of fields like the total current field that have no divergence are unusual [20]. They have no sources or sinks in two dimensional circuits. The streamlines of total current flow in loops and circuits wind up where they begin. The implication is that an (imaginary) particle of total current experiences forces all along its loop that depend on its eventual endpoint. The movement in streamlines is enormously correlated. Otherwise, the current would not flow in streamlines, in loops, i.e., in circuits. The flow of charges is anything except independent.

Calculations of the movement of such charges (and their displacement current) will not succeed if they only use the static Coulomb’s law without correlations as is usually performed in the simulations of molecular dynamics and elsewhere, as Feynman discusses at length ***in his entire*** Section 15-6 entitled “…statics is false for dynamics” [2].

The rules of current flow are known and true in nerve cells and mitochondria, as well as everywhere else. Biology is no exception from the laws of physics. Sometimes the complexity of biology makes the application of the laws of physics difficult. This is not the case with the Maxwell core equations. When the Maxwell equations are written without dielectric approximation, they contain no adjustable parameters. They do NOT depend on any properties of matter. They are true on all time scales that have been measured.

When current laws are derived by mathematical operations (Appendix B) without approximation or physical models (at all), they must be obeyed by biophysical models. There is no vitalistic exception allowed including that implied by the classical chemiosmotic theory [8,9,10,11,12] (because it does not mention Kirchhoff’s law or classical electrodynamics and thus ignores physical laws and the experimental reality of how currents actually flow). Modifications [8,9,10] begin to address the issues but do not use current laws and do not mention the laws of electrodynamics, as far as I can tell.

Not all biology has ignored the laws of current flow. Nerve and muscle fibers, and the syncytia of heart muscle, have long been analyzed as circuits with current flowing in complex biological structures [23]. Electrons transporting in chains and protons flowing in pathways in mitochondria also form circuits even though such circuits have not been analyzed with current laws in the chemiosmotic tradition [12]. Such circuit models can include coupled chemical reactions, diffusion, and even water flow. Quantitative predictions of current voltage relations have been made using a combination of current laws and chemical reactions in a wide range of conditions for complex 4 of the electron transport chain, cytochrome c oxidase [1]. The complexity of cytochrome c oxidase did not prevent a complete analysis and prediction of a wide range of variables under more or less any experimental condition.

Current flow generates the signals of nerve cells [23], muscle fibers, and the heart, as has been shown by some ninety years of work in biophysics. Currents link and correlate the properties of ion channel proteins to produce the signals of nerve cells. Without the correlation of the opening of ion channels, the nerve signal does not exist. The rules of current flow show how the propagation of the action potential depends on the correlations of channel opening at different locations [23], produced by the different potentials at those locations. The longitudinal current down the nerve—carried by whatever ions happen to be present, or even by electrons in a return path [24,25,26]—produces the different potentials that correlate the openings of various ion channels at different times, making the action potential a propagating wave form. The ability of electrons in a wire to support conduction of an action potential is a simple and striking proof of the role of current, ***not the flow of chemical species***, in the generation of a propagating action potential. This fact was discovered by Tasaki before the Second World War and rediscovered by Huxley and Stämpfli soon after the war [24,25,26].

The chemical approach to ion channels [27] is essential to understand each channel as a protein but it is not enough to determine how channels work together to produce signals. Ion channels in nerve signals are not linked by conformational changes or chemical reactions. The proteins are too far apart and too well shielded by ionic atmospheres and polarization, measured by Debye lengths and Bjerrum lengths, respectively [28].

Proteins are linked by potential changes produced by current flow [29] within the nerve cell, down the length of the nerve or muscle fiber. The conformational changes of ion channels are linked by electrodynamics in a natural nerve fiber. The proteins are not linked at all when potential changes and correlations are removed by a voltage clamp apparatus [30]. Indeed, the purpose of the voltage clamp system is to remove such linkages [31].

The forces that link conformational changes are nearly impossible to study atom by atom as is attempted in classical molecular dynamics. There are just too many charges involved to allow the evaluation of forces between atoms from the “potentials” of molecular dynamics. The properties of current that link the conformational changes cannot be computed from an electrostatic theory like the chemiosmotic hypothesis because it does not involve the only Maxwell equation that includes a current variable, the Maxwell Ampere law. It should be noted that molecular dynamics is not used to analyze electrical circuits in the physical sciences. The detail of atomic interactions is not computable, nor is it needed. Plasma physics has learned to deal with Maxwell’s equations in similar systems, at a variety of resolutions, but those methods have not been used in molecular dynamics, as far as I can tell [32,33,34,35,36,37,38,39].

Fortunately, the laws of electrodynamics extend beyond the laws of charges and their interactions. Maxwell’s version of Ampere’s law is labelled equation **A** in his summary paper—see pp. 465 and 480 of [40]. The ***Maxwell Ampere law provides extra information*** [38] that dramatically simplifies the analysis of many systems: only handfuls of currents are needed to analyze many circuits where a multitude of charges would be needed.

Interactions among currents are easy to evaluate using a corollary of the Maxwell Ampere equation, Kirchhoff’s law (Appendix B). Physicists and engineers have used Kirchhoff’s current law and its generalizations for some 150 years [41,42,43,44], even extending it to microwave frequencies [45,46,47,48,49,50]. Handfuls of currents are enough to analyze complex circuits that involve the movement of astronomical numbers of charges and beyond astronomical numbers of interactions of those charges.

Without using the rules of current flow, analysis of the correlations that create the signals of telegraphs, or computers, or our nerve cells, would be impossible. Calculations would have to evaluate the interactions of $>10^{10}$ charges. The number of combinations of $10^{10}$ charges, two charges at a time is $\left(10^{10}!\right)\left(\left(10^{10}-2\right)!\right).$ An enormous amount of coarse-graining would be needed to make such calculations computable, and the result would be inaccurate. As we shall discuss later, coarse-graining can be conducted by using current laws that are exact because they exploit other information beyond the charge interactions. ***The current laws provide an EXACT coarse-graining inherent to the Maxwell equations unlike other forms of coarse-graining which often involve unknown errors***. The current laws are mathematical corollaries of the Maxwell Ampere law, derived without approximation and are truly independent of models because the Maxwell Ampere law does not involve parameters of materials. It is as exact and universal as any known physics and applies to stars, ionic solutions, and mitochondria.

**Current Flow in Mitochondria**: In this paper, we discuss the rules of current flow in another membrane structure of biological importance: the electron transport chain of mitochondria. We use the tradition of analysis of ion channels supplemented with chemical reactions as shown in practical detail in [1]. This analysis includes the proton motive force and the movement of protons just as it includes the driving force and movement of all ions. This analysis creates an electro-osmotic theory that automatically includes the chemiosmotic hypothesis. It in fact uses the laws of current flow to simplify the traditional approach to the chemiosmotic hypothesis. It is difficult to see how a traditional chemiosmotic theory with its electrostatic restrictions (that preclude current flow) could deal with all of the inputs to ATP synthase. It is easy to see how the electro-osmotic theory provides the total current to drive the synthase.

The electron transport chain of mitochondria and chloroplasts creates the chemical energy of life that is stored in ATP. Applying the rules of current flow will help to analyze these important systems. Ignoring the rules of current flow would require charge-by-charge analysis. Charge-by-charge analysis would make quantitative analysis of the currents in mitochondria almost impossible just as it would make the quantitative analysis of nerve signals—or the circuits of electronic technology—impossible, and for the same reasons: there are too many charges and the currents change in nature from conduction to displacement continually if not continuously in such systems (Landauer, op.cit. [22], p. 112).

**Coarse-graining by Kirchhoff’s Law:** Impossibly large calculations of this sort are common in statistical physics. The impossible is often made possible by approximations that reduce the resolution of models. Approximations are necessarily made as coarse-graining is used to reduce complexity.

Indeed, analysis of currents—instead of charges—uses Kirchhoff’s current law to reduce the complexity of calculations (Appendix B). Far fewer currents are needed in Kirchhoff’s current law than charges are needed in Coulomb’s law, or the Poisson equation, or the continuity equation. Kirchhoff’s law seems to lower resolution as it replaces charges with currents. But Kirchhoff’s current law is an unusual coarse-graining because it introduces no errors. It is not an approximation. It is a corollary of the Maxwell equations.

Kirchhoff’s law, or rather its generalizations [38,41,51] are exact [52], Appendix B. It is not approximate. Indeed, the movements of all charges (at any time and location) can be calculated from Kirchhoff’s law or its generalizations because they are exact. ***Coarse-graining by Kirchhoff’s law is exact because it invokes additional physics, namely the Maxwell Ampere current equation***. Computation of time-dependent electrical forces cannot be performed using charge-by-charge analysis, as Feynman discusses at length in Section 15-6 entitled “…statics is false for dynamics” [2]: Coulomb’s law, for example, does not apply to time-dependent systems. Additional information is needed and that comes from the time-dependent properties of charge movement that produce magnetism. Current flow cannot be analyzed without considering magnetism because magnetism is driven by the velocity of movement of charges. The relative velocity of movement depends on the observer and so involves the theory of special relativity. Indeed, the theory of relativity was derived on this basis [53,54] as is well explained by Feynman in Section 13-6 of [2] and in magnificent detail in Ch. 12 of Griffiths [5].

The Maxwell Ampere law specifies properties of magnetism. ***Magnetism is not present in Coulomb’s law, the Poisson equation, or the continuity equation***. The Maxwell Ampere equation is what allows light to propagate through a vacuum devoid of charge. Coulomb’s law, the Poisson equation, and the continuity equation do not allow that by themselves. The special properties of magnetism **B**, specifically of **curl** **B**, determine the properties of currents and circuits, even if magnetic forces and energies are negligible in those circuits and currents. Current laws are derived from the Maxwell Ampere equation by mathematical not scientific operations that do not depend on the size of magnetic energy. ***Current laws do not involve material properties at all***. The only parameters involved are (any two of) the electric constant $\varepsilon_{0}$, the magnetic constant $\mu_{0}$, and the speed of light c. Coarse-graining by Kirchhoff’s law and its generalizations is a corollary of the Maxwell equations and is as valid as the Maxwell equations themselves (Appendix B).

The mathematical derivation of the current laws does not depend on approximations or models of material polarization and charge movement. This means that the coarse-graining is valid at all times and for all flows, in fact, whenever the Maxwell equations themselves are valid. Indeed, the physical and mathematical structure of the laws of electrodynamics force current to flow in streamlines and these form loops and circuits ***that neither begin nor end*** in two-dimensional or finite systems [20]. The only way total current can flow in streamlines is in circuits that obey Kirchhoff’s law or its generalizations. Models of biological systems must obey this physical reality if they are to avoid vitalism.

Many other coarse-graining procedures are more restricted. Some are in fact derived from equilibrium (zero flux) theories like statistical mechanics. Equilibrium theories cannot apply to systems far from equilibrium because they do not even include variables describing flow. Examples are electrical circuits, nerve signals, or electron transport chains of mitochondria [55]. Traditional chemiosmotic theories do not identify flow with variables or define electrical current as far as I can tell. ***It is difficult to compute something that has been assumed to be zero***. It is difficult to make a meaningful theory of reasonable sized flows that does not include velocity as a variable or friction (i.e., diffusion constant) as a parameter.

Sometimes equilibrium analysis can be extended to deal with small flows, but not without much effort. (Small flow analysis is not useful for engineering devices because the flows necessary to maintain their robust transfer functions are large. Such is the case for current flows in the ionic channels of nerve and muscle. They are large, nonlinear, and time-dependent, and classical small signal analysis is not useful). Asymptotic analysis of singular perturbation theory shows how small flows can be described more or less uniquely without evident internal contradictions [56], if sufficient care is taken. Singular perturbation theory also shows the ambiguities and contradictions that occur if the explicit analysis of that theory is not conducted particularly of conditions at intersecting boundaries. It also illustrates—in Section 2 of [56], particularly Figure 2.3.2 of this reference—the difficult ambiguities of systems containing multiple interacting parameters and fields, like the ionic solutions in which biology exists. These ambiguities are not discussed in the linearized Green Kubo theories of chemical physics as far as I know. They seem not to mention boundary conditions.

**What are the rules of current flow?** The fundamental rule of conduction current in circuits is a generalization of conservation of charge, called the continuity equation, described in every textbook of electrodynamics, e.g., Zangwill [4], p. 32, and Griffiths [3], pp. 222, 356. The conduction current is the current produced by the flux of mass with charge. The practical discussion in Ulaby and Ravaioli’s “Applied Electrodynamics”, Section 6.1 [57], is particularly relevant to the biophysics of patch clamp recording of single channels [58]. Following the practice insisted upon by Maxwell [41,42], Ulaby and Ravaioli explain that the current in circuits is not just the conduction current. The current in circuits is not just the movement of charges, electrons, or ions. **It includes the storage of charge (by displacement current at nodes of a circuit for example) as well as the movement of charge with mass**. It includes the special case of very small currents in very high impedance structures, which are so relevant to the biophysics of single ion channels [58]. Displacement currents play an important role in the study of single channels. Indeed, ***Maxwell said explicitly in the strongest possible language*** that ***ONLY*** computations involving true current (including the displacement current) could predict the movement of electricity [41,42]. Maxwell said this more than a century before the discovery of single channels.

**Displacement Current in Circuits.** Currents that actually flow in circuits and systems always include the displacement currents that accumulate to store charge [38,52]. Displacement is the name for currents that depend on the time rate of change in potential ∂V/∂t. The name “displacement” emphasizes the importance of time dependence in the process. Charge must move, i.e., be displaced, to create displacement current. The steady movement of charge (in which the time rate of change in the electric field ∂E/∂t is zero) does not produce displacement current.

The displacement current has the special property that it is zero when the perturbing potential is zero and steady. The displacement current is zero when nothing is displaced, when potential does not change with time. The displacement charge returns to its starting place when the perturbation ceases. All of the charge that is displaced returns. The displacement current returns to zero when the perturbation ceases. Charge is conserved. No charge or current leaves the system. No steady current flows in such systems, so there is no steady energy loss to friction.

Displacement currents have been extensively studied in biophysics because they are an essential part of the process that produces signaling in nerve cells. The special properties of displacement current—the charge displaced at the ON of a rectangular voltage pulse equals the charge displaced (actually charge replaced) at the OFF—have allowed measurement of nonlinear voltage-dependent gating currents in muscle [59] and nerve [60] that arise in special structures in some channels called voltage sensors [61,62]. The equality of ON and OFF displacement charge has not been used so far in the construction of energetic functions for polarization [19].

**Displacement Current in Biology.** Biologists discovered the importance of displacement current in the 1930s. Capacitive displacement current was discovered as biologists studied the signals of the nervous system and then muscle and the heart. At first, biologists—including the Nobel Laureate Chair of Biophysics A.V. Hill [63]—sought a biochemical explanation of the action potential and its propagation in the flux of a chemical species, driven by diffusion—but the elegant experiments [64,65] of the Cambridge physiology student A.L. Hodgkin, followed by Tasaki, then Huxley, and Stämpfli [24,25,26] showed that propagation is an electrical phenomenon that does not depend on the flux of a chemical species. Rather it depends on the flux of an abstract quantity unique to electrodynamics, namely the electrical current. Chemical propagation of the action potential does not occur. Indeed, ***Tasaki showed that the conduction of electrons in a wire could support propagation of an action potential*** before the second world war!

Hodgkin [66] and Huxley’s [23] later work showed that electrical analysis gave a quantitative understanding of the nerve signal, while the biochemical explanation of propagation—then and now [27,67,68]—is qualitative. Biochemical understanding is enormously important of course, but qualitative nonetheless. It cannot compute the shape of the propagating action potential, or even the conduction velocity, for that matter. Prediction requires numbers to describe potentials because the action potential is a phenomenon, a signal, a waveform distributed in time and space. The action potential is a set of numbers, neither the single number meant by the word “potential” in physics, nor a phrase without numbers as meant in biochemistry. The biochemical treatment of channels is verbal and qualitative. It does not produce numbers. Theories that do not involve numbers, like the chemiosmotic theory, cannot produce quantitative predictions and understanding of physical or biophysical phenomena. The chemiosmotic hypothesis in original form is not a theory for that reason. The electro-osmotic approach is struggling to become a full theory. Work on cytochrome c oxidase shows that the electro-osmotic approach is quantitative, general, and feasible, but the theory has not been applied more generally than that [1].

**Molecular dynamics** extends the chemical approach and does produce numbers. But it cannot predict the coupling of sodium and potassium channels in the action potential for a technical reason. Coupling in the action potential is produced by a long-range change in potential that is not included in the simulations of modern molecular dynamics. These simulations keep track of the movement of ions, without considering long-range electric fields such as those that spread down the nerve fiber. Long-range fields involve long-range current flow that involves staggering numbers of ions and their interactions as we have already discussed.

Long-range fields cannot occur in the periodic systems studied in molecular dynamics. In periodic systems, potential cannot spread beyond the length of the period. Indeed, the potentials at both ends of the period are the same, so the potential distribution within the period is controlled by an artificial periodic condition not found in the original system before simulation. ***Artificial boundary conditions produce artifacts*** at boundaries. Membranes are boundaries used by biology to perform many essential functions of life. Periodic boundary conditions produce artifacts at the very place that produce crucial biological phenomena, like action potentials or ATP in nerves and mitochondria, respectively.

Classical molecular dynamics also does not deal with the capacitive, displacement current. It uses Coulomb’s law to evaluate time-dependent forces (despite Feynman’s emphatic objection that ***“Coulomb’s law is false in dynamic systems”*** [2], Section 15.6). Both these issues have been dealt with in plasma physics with particle or even atomic resolution [32,33,34,35,36,37,38,39]. Particle-in-cloud methods [34] have also been applied to discrete models of current flow in semiconductors for a long time, e.g., see application to **PNP** transistors [69,70]. These methods seem not to have been applied to molecular dynamics of ionic (i.e., electrolyte) solutions or proteins. “Plasma” in physics is different from “plasma” in ionic solutions. In physics, plasma refers to a gas of ions, not the dissolved ions of blood without cells. Ions in physical plasmas often have negligible diameters. Ions in electrolytes have finite diameters that are of great importance in determining the selective (non-ideal) properties of ions crowded into channels and enzyme active sites and in concentrated bulk solutions [71].

Analysis based on the properties of current flow—***not*** the properties of movement of individual charges—forms a quantitative description of nerve signals. Hodgkin and Rushton [72] showed that excitable cells could be analyzed with nearly the same theory that Kelvin used [43,44] to analyze the Atlantic cable, even though electrons carried the current across the Atlantic and ions carry the current down nerve fibers. Hodgkin and Huxley [29,31] used Kelvin’s cable theory to analyze the nerve signals discovered by Volta and Galvani. Tasaki, then Huxley, and Stampfli showed how electrons could carry return signals in myelinated nerve fibers [24,25,26]. Ulaby and Ravaioli have an extended discussion of such cables, transmission lines, and telegrapher’s equations in a modern context [57].

In nerve cells, one set of ions carries a chemical flux of ions through channels across the membrane, carrying charge and creating a conduction current as it moves. Another set of ions altogether carries the current along the nerve cell, creating propagation [23]. It is the current flow—not the chemical flux of one type of ion or other—that produces propagation as shown directly by measurements on axons perfused with different solutions [73,74]. The longitudinal potential gradient depends only on electrical properties. It depends on the resistance per unit length of ionic solution inside the cell, not on frequency or time even in cells with cytoplasm filled with proteins [75], and not on the composition of the solution. The change in concentration produced by the chemical flux has no role in the Hodgkin Huxley analysis of propagation [29,31]. The current down the axon changes the potential across all molecules in the membrane. It is the propagating potential accompanying the longitudinal current that correlates the opening of the channels. The channels are separated. They are quite distinct, molecularly independent proteins selective for sodium or potassium ions.

(Historical note: The idiosyncratic, if not chauvinistic, operator mathematics of Hodgkin and Rushton was replaced by more customary mathematics in [76,77,78]. Ref. [79] uses the modern two-port theory that allows easy combination of different transmission lines in parallel, series, or almost any other arrangement. Most analysis today is performed with software packages which are easily located by searches for “applied electrodynamics software”).

**Membrane Capacitance:** The capacitive current through the cell membrane played a crucial historical role in validating the voltage clamp analysis of the action potential (personal communications from A.F. Huxley and separately A.L. Hodgkin to the author, in the 1960s). The formula for the displacement current $C_{m}\partial V/\partial t$ in Hodgkin, Huxley, and Katz [30], Equation (11) (describing the axial wire setup of Figure 10), allowed analysis of the ionic current when the voltage was changing, for example, during an action potential. They needed to study the current during the natural behavior when the voltage was changing during an action potential, when ***the voltage clamp amplifier was not present***. $C_{m}$ is the membrane capacitance and $\partial V_{m}/\partial t$ is the time rate of change in the membrane potential. The axial wire used by Hodgkin, Huxley, and Katz [30] removes the current down the axon and prevents propagation but in itself it does not control the time-dependence of the voltage. That is a separate function, performed by the voltage clamp amplifier. The axial wire forces the total current (capacitive plus ionic) across the membrane to be zero [30], Equation (11). In that case, $C_{m}\partial V/\partial t$ equals the total ionic current $\sum I_{j}$ across the membrane. j identifies the type of ion, typically ${Na}^{+}$ or $K^{+}$.

**Potentials are uniform in short cells.** In fact, the same HHK equation ${\sum I_{j}=C}_{m}\partial V/\partial t$ describes ***ANY SYSTEM*** in which all membranes/ion channels/transporters have the same transmembrane potential across them [80,81]. In any such system, the capacitive current equals $C_{m}\partial V/\partial t$ is equal in magnitude to the total ionic current $\sum I_{i}$ but in the opposite direction across the membrane. The mathematics establishing this result for long and short cells is derived in [81] and explained in textbook detail in [56], pp. 218–238. Errors in the approximation (for short cells) are calculated explicitly. Short cable theory is discussed in many texts and other references, e.g., [82,83].

**Figure 1:**
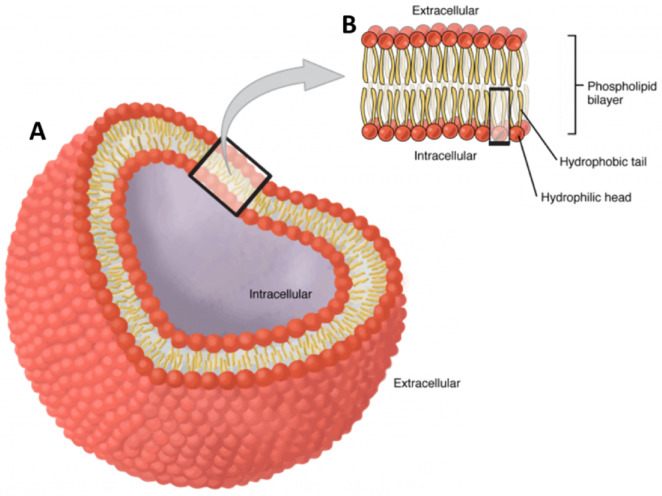

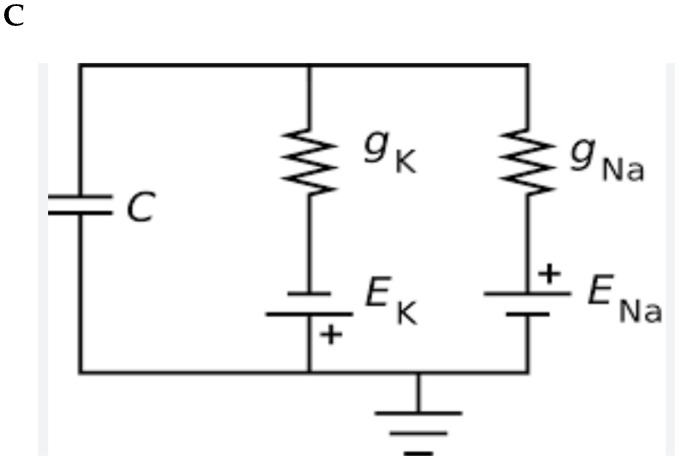
(**A**) A short cell with (**B**) a lipid bilayer membrane. Ion channels are not shown. (**C**) The currents across a short cell. The classical Hodgkin Huxley system is shown of a membrane capacitance C, with a current pathway for $K^{+}$ ions through a potassium conductance $g_{K}$ driven by the gradient of chemical potential $E_{K}$ and similarly for sodium ${Na}^{+}$ ions driven through a sodium conductance $g_{Na}$ by the gradient of chemical potential $E_{Na}$. The sum of the currents through all three pathways is zero. There is no other place for the current to go! The HHK equation ${\sum {\;I}_{j}=C}_{m}\partial V/\partial t$ applies to a short cell or organelle of irregular shape like a mitochondria, as long as all of the membranes, channels, and transporters have the same potential across them.

In words, without mathematics, short cells (and organelles) have (nearly) uniform internal potential. Only cells with long processes like axons and muscle fibers have non-uniform transmembrane potentials. Short cells and organelles have only one transmembrane potential (see Figure 1), so the total ionic current $\sum I_{j}$ through the membrane equals the capacitive current $C_{m}\partial V/\partial t$.

(1) $$\mathrm{HHK}\;\mathrm{equation}:\;{Sum\;of\;All\;Ionic\;Currents=\sum {\;I}_{j}=-C}_{m}\partial V/\partial t$$

$I_{j}$ **describes *any* current** carried by ions with mass including those carried by electrons, or protons, through active transporters or channels. ${\;I}_{j}$ is not restricted to the currents through the Hodgkin Huxley ionic conductances identified in Figure 1.

**HHK Equation and Kirchhoff Coupling:** Equation (1) is called the HHK equation because it appeared so clearly in [30] in Equation (11) and was so important in their analysis as a check on the voltage clamp results [29]. The HHK equation expresses the coupling produced by Kirchhoff’s current law in any system characterized by a single transmembrane potential. A version of the equation describes the principle “you must complete the circuit” known to telegraphers for a very long time, roughly since 1840.

The HHK Equation (1) implies a coupling between currents that is driven by electrodynamics, not by chemical interaction. If one current increases, some others must decrease. A graph of one current vs. the other (with everything else fixed) will show a straight-line 45-degree dependence. That is an operational definition of coupling that can be applied to any experiment measuring currents or fluxes, whatever the origin of the currents, including the proton currents driven in part by the proton motive force. This definition applies to experiments. It does not depend on models. In the setup of Equation (1), the electric field and electrical potentials change fluxes in exactly the amount needed to create coupling.

**Figure 2:**
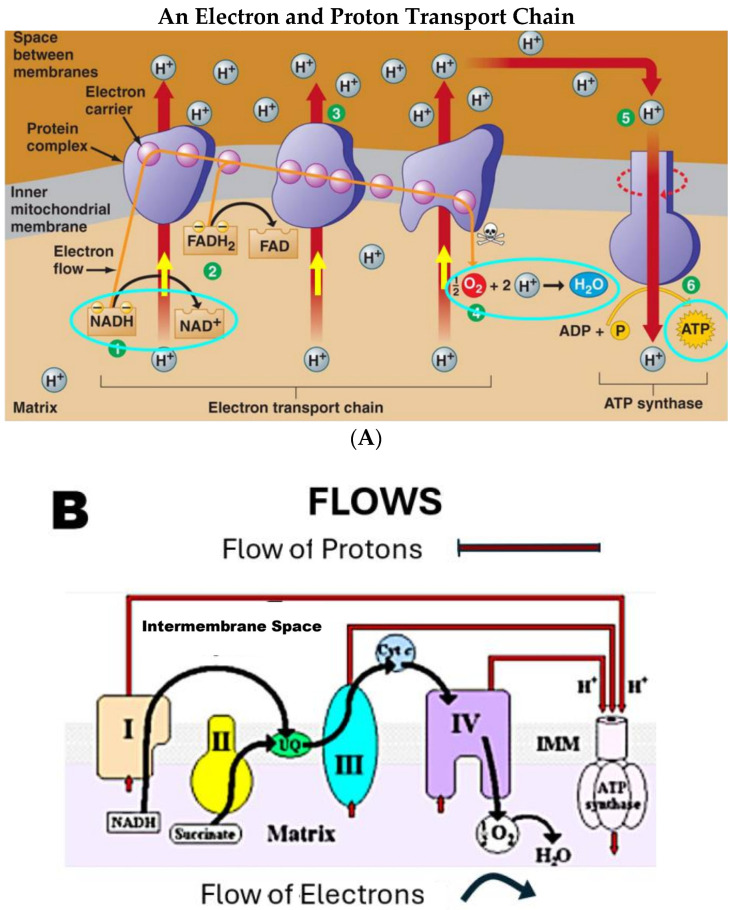
(**A**) A schematic view of a mitochondrion emphasizing the functionally important roles of the four complexes identified by the numbers 1, 2, 3, 4, 5, and 6 enclosed in green circles. ATP synthase might be called a fifth complex: it gathers the currents of the other four complexes to drive the actual synthesis of ATP. The main function of these complexes is to provide the “flow of protons” visually defined as a brown bar (within black borders) at the top. (**B**) The four complexes are identified by the Roman numerals **I**, **II**, **III**, and **IV**. **The flow of electrons is shown as a solid black curved thick arrow as visually defined at the bottom of** (**B**). The spatial organization of the complexes is beyond the resolution of the analysis of this paper and surely will require further analysis.

The Maxwell equations change the potential to create the coupling. It is the NON-constant field [84,85] that creates the coupling. Insistence on constant fields makes this coupling hard to understand [86]. The importance of NON-constant fields is not confined to biophysics. It is in fact central to circuit theory in general. Ref. [21], Figure 2, shows how the microphysics of individual devices accommodates (and enforces) the relations of currents demanded by the Maxwell Ampere equation. It is fortunate that semiconductor physicists abandoned the idea of a constant field (compare [87,88] and [89,90]; see [91] for a modern treatment). It is difficult to imagine Shockley’s understanding [92,93,94,95] of transistors (both bipolar and field effect) emerging from a theory based on constant fields.

The coupling implied by Equation (1) is universal—is as universal as the Maxwell equations of electrodynamics [38,52]—because it arises as a corollary of the Maxwell Ampere partial differential equation. The coupling occurs between widely separated atoms. It can be remote on the length scale of Kelvin’s undersea cable of thousands of kilometers or on the atomic length scale of angstroms: It occurs in systems screened by ionic atmospheres (measured in Debye lengths) and induced polarization charge (measured by Bjerrum lengths), both a few angstroms in size in most biological situations.

The HHK Equation (1) includes displacement current following Maxwell’s admonition [41,42]: **“ … that the time-variation of the electric displacement, must be considered in estimating the total movement of electricity.”** Quotation and supporting equations are in Volume 2, Section 610, p. 232. ***Maxwell could hardly have chosen more emphatic language***. He calls the total current “the true current of electricity” …”that must be considered in estimating the total movement of electricity.” He implies that the neglect of true current (with its displacement current component) will not allow prediction of the total movement of electricity. Maxwell illustrates the general principle with examples and applications calculated in detail, that show in detail how correct prediction requires correct treatment of displacement current lest his admonition be viewed as a remote abstraction irrelevant to practical applications.

I call the coupling implied by the HHK equation “Kirchhoff coupling” to emphasize its electrical nature. It might be better called “Maxwell coupling” [41] when the displacement current (like that of the membrane capacitance) is important (Appendix B). The phrase “Maxwell coupling” emphasizes the dynamic aspect of coupling described vividly in Feynman’s admonitions ([2] Section 15.6) not to use Coulomb’s law when electric fields vary with time. Coulomb’s law might be an adequate approximation for slowly changing systems, but its uncritical use obscures the underlying physics, even when it is an adequate approximation. Lest this be viewed as the idiosyncratic view of the author, one should consult the many pages in which Feynman says that Coulomb’s law is NOT true for a time-varying system.

**“Protons” in Biology:** Note that any of the positive charged versions of water—customarily called “protons” or $H_{3}O^{+}$ in the chemistry literature [96]—all count as one of the types of ions in Equation (1). Proton currents are tricky to deal with, because several forms of hydrated protons exist [96]. Each can participate in a wide variety of protonation reactions with various substrates as they move as a current. Proton fluxes must be analyzed by electrodiffusion equations in conjunction with the chemical reactions that increase or decrease proton concentration as substrates protonate or deprotonate, as is conducted in [1].

The proton current needs to be analyzed by the theory of complex fluids because it involves so many different forces and fields. ***These fields are not described by the theory of simple fluids*** or by chemical reaction theory that does not deal with electrodynamics, diffusion, or friction. Composite theories tend to be inconsistent if they are not derived using complex fluid theory, particularly in its variational formulation [97,98,99]. Inconsistent theories produce different results in the hands of different scientists.

We turn now from current flow in nerve and muscle to current flow in mitochondria and chloroplasts.

**Mitochondria and Chloroplasts:** Mitochondria are the powerhouse of animals, closely related to chloroplasts which are the powerhouses of plants as they create oxygen gas and thus allow animal life. Both generate the ATP that stores chemical energy in both plants and animals. The mechanisms involved have been a central subject in biology for a very long time because they are a keystone in the arch of life. Without ATP production, the arch of life collapses, and both plants and animals die. Life requires the hydrolysis of ATP.

ATP, crucial to life, is generated in mitochondria shown in Figure 2 by a set of protein complexes numbered Figure 2A and shown in Roman numerals I to IV in Figure 2B. The protein complexes are spatially separated in the mitochondrial inner membrane [15,100,101]. The outer mitochondrial membrane is not directly involved because it has quite low resistance and does not change current flow very much.

From the low-resolution point of view of this review, the cristae and complex folding of the inner membrane [102,103] are not important so long as all of the membranes have the same transmembrane potential. The function of the complex structure will likely emerge in higher-resolution analysis dealing with variation in transmembrane potential, just as the complex structure of skeletal muscle emerged as the function of the transverse tubular T system was discovered [104,105].

The importance of complex structure is not to be ignored just because the present form of the electro-osmotic hypothesis does not include it. There are of course a hierarchy of structures involved in the function of these biological systems [106,107,108,109], with both the spatial organization of mass and the spatial organization of current flow being important.

The spatial organization (anatomy) of mass does NOT identify the electrical organization. The anatomical structure constrains the pattern of current flow, but ***the distribution of ion channels (and pumps and membrane capacitance) is at least as important as the spatial organization***. In particular, to deal with the substructure of the complexes, one must know what fraction of the membrane potential of the mitochondria appears across each of the circuit elements of the complex.

The analogy with skeletal muscle is clear. The different ELECTRICAL organization of the T tubular system and the Sarcoplasmic Reticulum (SR) produce totally different functions, one as an extension of the electrical signaling system (T system) and the other as an electrically ISOLATED calcium storage system (SR).

Sadly, there is almost nothing known about the spatial organization of current pathways and so a treatment in this paper would be premature.

From a chemical perspective, the generation of ATP depends on metabolites like NADH (nicotinamide adenine dinucleotide) that store electrons. The stored electrons produce high-energy molecules poised to be electron donors when they participate in the appropriate chemical reactions. Evolution has designed those metabolites and chemical reactions, so they provide functions useful to the survival and reproduction of life.

The high-energy molecules drive the transfer of electrons from donors to lower-energy molecules in a sequence of ***spatially separate*** oxidation–reduction reactions moving electron charge from one intermediate to another.

**Figure 3:**
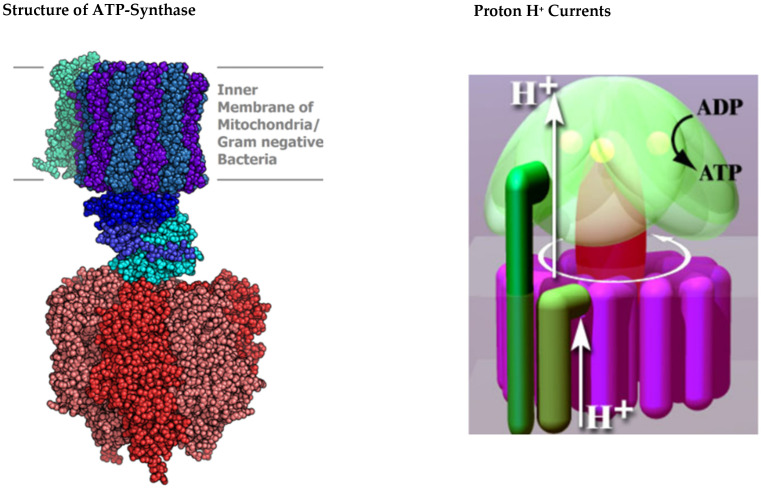
The structure and proton H^+^ currents in ATP synthase. The proton movements drive the synthesis of ATP from ADP by the rotation of the electrostatic turbine. The current flow of protons H^+^ can be analyzed—as can any other current—by Kirchhoff’s current law and its generalizations because they are corollaries of the Maxwell Ampere law of electrodynamics. The corollaries are derived without physical models or mathematical approximations. In particular, the current flow of protons H^+^ through ATP synthase obeys the HHK Equation (1) in a short system with a single transmembrane potential. In a short structure like a mitochondrion, the sum of that current and all other currents is zero: everything that flows in, flows out.

Entropic losses are decreased by the use of a sequence of reactions, reducing lessening friction and heat generation in each one of the processes. More energy is then available for other cellular functions. The electrical energy moving in the electron transfer chain is ultimately provided to ATP synthase (Figure 3) in the form of currents of protons.

Electrons moving from one location to another form an electron current whether those electrons flow in a mitochondrion or Kelvin’s submarine cable or in anything else. Electron currents have been analyzed by Kirchhoff’s law from long before the discovery of the electron because the law gives a simple successful description of what is seen in experiments.

**ATP Synthase:** Protons eventually drive the synthesis of ATP in the extraordinary enzyme ATP synthase shown in Figure 3. ATP synthase is not mechanically linked with the electron transfer complexes. It does not share conformational changes or oxidation–reduction (redox) metabolites like NADH for FAD. Rather, ATP synthase is an electrical turbine that uses the flow of protons to perform rotational catalysis. Electrical analysis involving numbers and equations is needed to analyze the flow of charges like protons from elements of the electron transport chain to ATP synthase. The flow of charge is the current of Kirchhoff’s law (Appendix B). Treating current flow without the laws of electrodynamics means violating the laws of physics. Current flow is not just the movement (i.e., the flux) of one type of ion. It has laws of its own derived from the properties of magnetism (the Maxwell Ampere law) that are not contained or implied by the laws of electrostatics or the movement of ions. For example, total current is never accumulated on any known time scale. The flux of individual species (e.g., protons) is accumulated on all time scales.

Rotational catalysis is performed in ATP synthase as the gamma subunit of the synthase spins in a revolving cycle of conformational changes. The rotation allows a different subunit (beta) of the synthase to position the substrates ADP and inorganic phosphate so they can react. The substrates join (“condense” in chemical language), forming ATP. **The mechanical energy of the rotation drives the synthesis of ATP**. The mechanical energy is derived from the flow, i.e., current of protons. The ATP is released when the beta subunits reach their open conformation.

ATP synthase can be viewed as an electrostatic counterpart of the DC electromagnetic motor of our technology. Permanent charges of the synthase play roles rather like the permanent magnetic stators of DC motors. They are structural catalysts that do not directly contribute energy to the process but make it happen, nonetheless.

**Protein Complexes of the Respiratory Chain**: We now turn to a brief discussion of the individual protein complexes [15,100,101,110]. The discussion and figures are meant to show that ***electron and proton currents are also the language used in the existing (experimentally oriented) literature of the respiratory chain***. Experimental work shows the importance of movement and flux. What the experimental work does not do is to use the powerful existing knowledge of that current flow developed by physicists since roughly 1850. Experimental work has neglected the universal properties of current flow. It has tried to use a STATIC view of charges to describe the dynamics of the currents that are so vividly illustrated in the illustrations of the experimentalists themselves that are reproduced here. Electrodynamics cannot be derived from electrostatics. That is exactly why Maxwell had to change Ampere’s law. Current flow in mitochondria cannot be analyzed by electrostatics.

Electron and proton currents are important inside each component of the respiratory chain, as well as in the conjunction of their currents shown in Figure 1. The conjunction of currents drives ATP synthase, as shown in Figure 2. The Kirchhoff law of current (and its generalizations) must be used to describe the charge movement that is so well illustrated in the experimental literature (Appendix B). ***There are no vitalist exceptions granted to the chemiosmotic theory***. The verbal vagueness and lack of quantitative analysis in the chemiosmotic hypothesis, particularly in its original form, should not be allowed to hide the fact that mitochondria are subject to the same laws of electrodynamics as everything else.

The charge movements of the respiratory chain arise in different locations. They interact and sum to create the net flow into ATP synthase as shown in Figure 2, particularly Panel B. The flows combine according to specific physical laws, e. g., the HHK Equation (1) Those laws include currents driven by diffusion and electrodynamics as well as electrostatics and chemical reactions.

The role of electrostatics in the respiratory electron transport chain is increasingly recognized. References [111,112,113,114] can serve as an introduction to that literature. But the literature seems not to describe the transport of electrons, protons and charge as currents satisfying the Kirchhoff current law (Appendix B). As Reference [114] puts it: when dealing with one crucial part of the respiratory chain, “Although the existence of the coupling between the electron transfer and the proton transport (PT) is established experimentally, its mechanism is not yet fully understood at the molecular level”.

This paper shows that much useful analysis is possible using the laws of current flow ***without*** full understanding at the molecular level. In the engineering and physical sciences, circuit analysis exploits the properties of current flow without understanding the details of the underlying charge movements. Similarly, the motion of every atom is not needed to understand many of the properties of the electrical and electronic circuits of our technology. Study of the details of charge movement is conspicuous by its absence in the encyclopedic treatment of electronic circuits of Horowitz and Hill [115]. In biological circuits, the motion of every atom is not needed to understand the mechanism of action potential propagation. Analysis of the electron transport chain may benefit from this type of approach. Such an approach is feasible [1].

The complex substructure of the electron transport chain [102,103] will add function no doubt, and result in substantial changes to this early form of the electro-osmotic hypothesis, but the more realistic complex version will involve the distribution of current flow according to the laws of electrodynamics just as the present early version of the theory does. The laws that govern current flow are universal.

**The Respiratory Chain**: Figure 2 shows the role of current in the entire respiratory chain. The figures in this paper show that each component of the respiratory chain involves current flow. These figures, taken from the existing public domain literature, are chosen to vividly illustrate the importance of current flow. The figures are not my original contribution beyond Ref. [1]. They are here to show that the idea of flow already permeates the literature. The contribution of this paper is to say those flows can be analyzed by Kirchhoff’s law and its generalizations (Appendix B). Kirchhoff coupling provides an important mechanism correlating the function of the protein complexes of the respiratory chain. That mechanism cannot be analyzed without dealing with current laws because too many charges are involved to allow the practical use of Coulomb’s law, Gauss’s law, or the Poisson equation. Current flow appears in only one of the Maxwell equations of electrodynamics, namely the Maxwell version of Ampere’s law that deals with magnetism. The laws of current flow are a consequence of the properties of magnetism. Electrostatics is silent about magnetism and therefore cannot deal with current flow. Adjunct models of current flow (such as the derivation of the continuity equation from flows in textbooks like [5]) have to be modified to deal with the total current. Indeed, that is precisely why Maxwell had to modify the original version of Ampere’s law.

Online videos provide particularly vivid (and beautiful) illustrations of the flows of electrons, protons, and charged groups. See particularly those of the BioVision group at Harvard, available on YouTube when this paper was written (https://www.youtube.com/watch?v=LQmTKxI4Wn4&t=324s, accessed on 1 June 2025).

**Figure 4:**
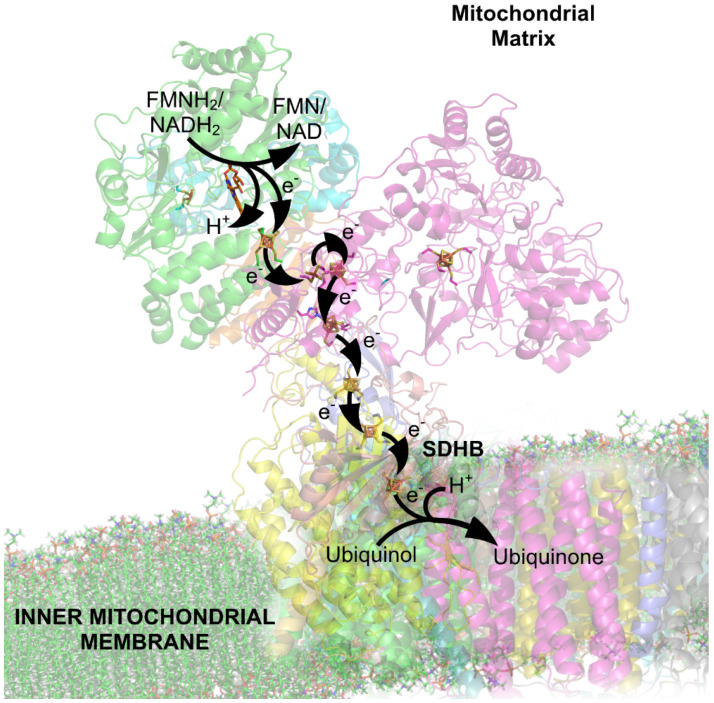
**Complex 1:** NADH dehydrogenase. The figure shows the long path for electron currents, >20 Å. FMN and FMNH_2_ are flavin mononucleotides. NAD and NADH_2_ are nicotinamides. SDHB, sometimes called SDH, is a subunit of succinate dehydrogenase as defined in the extensive literature of that enzyme. The intramolecular flow of electrons obeys (quantitatively) the Maxwell equations, including the generalized Kirchhoff current laws, and thus depends on other pathways for current flow (Appendix B): the HHK Equation (1) and Figure 2B. That dependence is not evident in classical expositions in the chemical tradition.

**Complex 1** is an NADH dehydrogenase [116] that transfers two electrons from NADH to a lipid-soluble carrier, ubiquinone, pp. 835–837 of [100,117,118,119]; see Figure 4. The reduced product flows through the membrane according to the laws of electrodiffusion in a coupled reaction, described in detail in the literature [100,117,118,119]. Complex 1 moves four protons (H^+^) across the membrane, producing a proton flow that will later be used to generate ATP, mostly through ATP synthase. Each transfer and flow are an electrical current that can be analyzed by electrodynamics, using Kirchhoff’s current law (Appendix B). Currents on this scale have been analyzed this way in ion channels for a long time: the structures in ion channels that control current flow are often only a handful of angstroms long.

**Verbal Description of Kirchhoff Coupling:** The HHK Equation (1). provides the information needed to understand the interaction of currents by Kirchhoff coupling. A verbal description of the implications of the equation may also be helpful: The potential in the mitochondrion adjusts itself so all of the currents that flow into the mitochondrion also flow out. There is nowhere else for the currents to go. The current through one component of the electron transport chain will thus interact with the current of any other component, because of electrodynamics, independent of chemical interactions.

It is important to remember that this Kirchhoff coupling is independent of the nature of the current flow. It only depends on the total current itself as specified by the right-hand side of the Maxwell Ampere law [7]. Of course, the Kirchhoff coupling does not conserve the flux of individual ions. All of the current that flows in must flow out. It is NOT true that all of the flux of a particular chemical species that flows in must flow out. Or more precisely, Kirchhoff’s law does not guarantee that. Different time scales and different transport proteins are involved in the processes that eventually balance out the fluxes often on a time scale of minutes or hours in biology (whereas the functions themselves are faster than seconds). Other laws and equations are needed to describe the accumulation of particular chemical species. Kirchhoff coupling occurs on all time scales, however fast (when true current is used) (Appendix B). At any instant, the total true current that flows in flows out, as long as the word current includes the displacement current. The Maxwell generalization of Kirchhoff’s current law holds at any instant of time. It is not an average or integral law. It is true on any time scale, no matter how fast, that the Maxwell equations themselves are true.

In contrast to current, flux does accumulate on a range of time scales. Flux accumulation may take a very long time. All of the flux flowing in may not equal the flux flowing out on many important time scales. The flux laws are integral laws that are true when integrated over long enough times, in contrast to the current laws which are “instantaneously” true. The fluxes usually eventually balance out. They are often driven by separate mechanisms not considered here. The mechanisms considered here do not balance out the integrated fluxes. Of course, the mechanisms of the action potential also do not balance out the integrated fluxes. After one action potential, there is slightly more sodium and less potassium concentration inside the nerve than before the action potential. There is a significantly larger increase in potassium just outside the nerve and that extra potassium can build up after a few action potentials and have significant effects. A separate mechanism, Na-K ATPase, eventually restores the concentrations.

The classical chemiosmotic hypothesis [8,9,10,12,111,112,113,120] does not deal with the balance of currents because it does not include equations and numbers. ***Verbal analysis does not allow quantitative balance***. As just explained, physics in general shows by quantitative mathematical analysis that the balance of currents is instantaneous, occurring on the fastest time scales; the diffusional and chemical interactions may be slow, in principle, even slower than the time scale of ATP formation by ATP synthase. ***It should be emphasized that mitochondria themselves follow the laws of electrodynamics, even if the classical chemiosmotic theory does not***. Mitochondria follow the current laws that come from the Maxwell Ampere law of magnetism. The chemiosmotic theory involves only charges and electrostatics and ignores the current laws that arise from magnetism and its properties.

Kirchhoff coupling has many names. All are logically equivalent descriptions of the HHK Equation (1). I list some here because experience shows that different names are used by different scientists, who may not realize the names are all equivalent, i.e., they describe the same HHK Equation (1).

One current is driven by the sum of the other currents.

Currents are correlated.

Currents interact.

The cause of one current is the sum of the other currents.

**Short and Other Structures**: The discussion applies to current flows in a short structure like a mitochondrion. If the components of the electron transport chain are not in a short structure, the interactions will be different. In particular, if the components are in a lipid bilayer, with a controlled voltage across the bilayer, the currents of the components will not sum to zero. Currents will not interact by electrodynamics and so the results will be different from the results measured in a mitochondrion. ***A chemiosmotic theory without numbers or equations will have difficulty explaining the difference in the interactions of components in short and long systems***.

**Figure 5:**
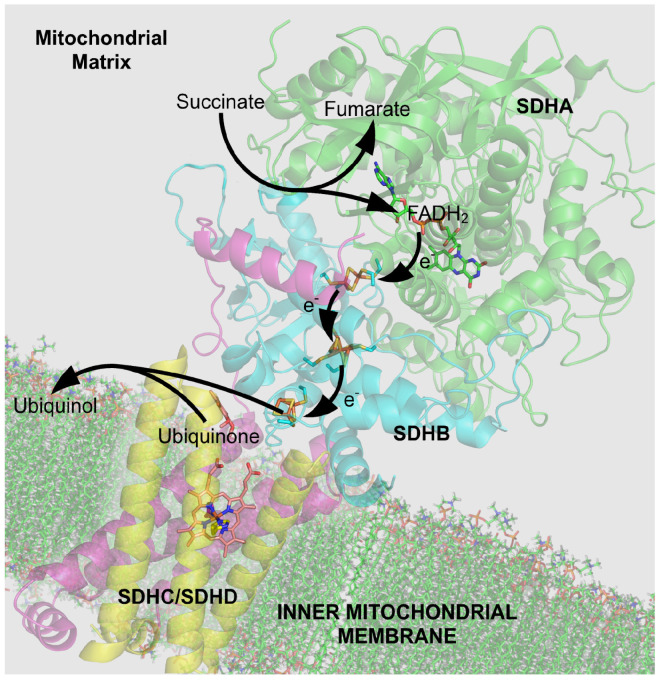
**Complex 2**: succinate dehydrogenase complex SDH. Notice the long pathway for electron transfer and current flow that must be described by the Maxwell equations for current flow including the HHK Equation (1), The current flow will interact with other flows in the system and so be different in measurements from isolated systems compared to mitochondria.

**Complex 2** (Figure 5) includes succinate-coenzyme Q Reductase [121] and participates in both the citric acid cycle of cell metabolism and the electron transport chain. Only electron flow occurs in this complex. Proton flow is not involved. Complex 2 contains a succinate-binding site and ubiquinone-binding chain of oxidation–reduction (redox) centers that extend over 40 Å providing an extended path for electron flow often called electron tunneling. The extended path may be partially within the electric field of the membrane. The electrical potentials driving electrons down that path are not clearly stated in the literature that I know of. Are those potentials the potential inside the mitochondria or outside the mitochondria, or some combination? What specifies the combination if it exists? Obviously, the amount of electron transport (i.e., current) will depend on the potentials driving the current through the chain of redox centers. Clearly, quantitative models and equations are needed to deal with these issues.

It should be clearly understood that the Maxwell equations—and the corollary current laws—apply to all current flow and thus electron transfer, whether by tunneling along an iron sulfur (Fe-S) chain or any other mechanism. See Appendix A and Appendix B. The current laws are truly independent of the electrical potentials driving them, just as the Kirchhoff law of currents in circuit analysis is true whatever the Kirchhoff voltage law says about the electrical potentials. The current laws of Maxwell remain valid in the quinone transfer chain just as they are in the delocalized electron orbitals of the wires in our computer circuits. They remain valid within the chemical domain described by the Schrödinger equation as shown in the general gauge invariant derivation in [38,52].

The movement of electrons will interact with other current flows across the mitochondrial membrane according to the HHK Equation (1) of this paper, as discussed at length near that equation, because that equation is a corollary of the Maxwell equations themselves.

It is important in actual practice that ***current laws do not depend on the description of individual charges***. It may be much easier to determine currents than charge flow in the various models of super complexes [122]. Interactions thought to depend on exact atomic geometry may well arise simply from the interactions enforced by the HHK Equation (1). That is in fact how currents in general interact in circuits, as explained in detail in Ref. [21]; see Figure 2. Note again that measurements in isolated systems will give different results from those in mitochondria.

**Figure 6:**
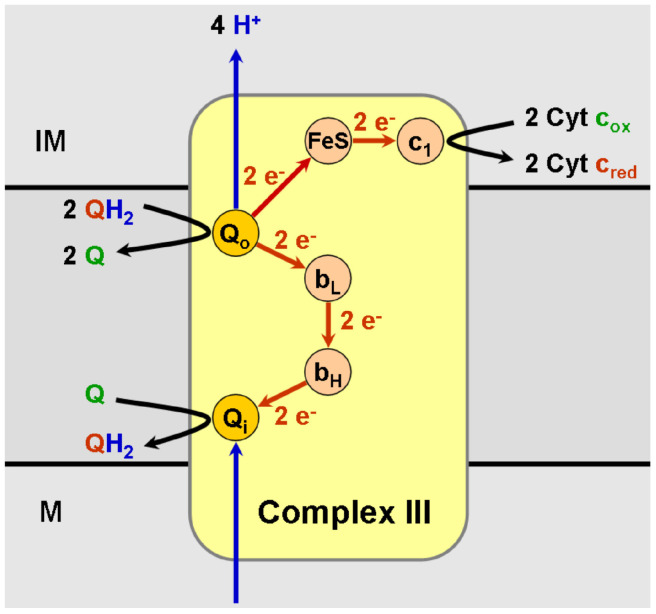
**Complex 3**, also known as coenzyme Q. The coenzyme transfers 4 protons long distances as shown by the vertical blue arrows and so provides an electrical current described by Kirchhoff’s current law (Appendix B). The current depends on all of the currents across the mitochondrial membrane as shown in HHK Equation (1). Q is the ubiquinone form of CoQ, and QH_2_ is the ubiquinol (dihydroxyquinone) form. Substates in circles are identified as parts of the Q cycle. FeS is an iron sulfur protein. Q_0_ and Q_i_ are ubiquinol (QH_2_) and ubiquinone (Q), binding sites, respectively. b_L_ and b_h_ are heme groups and c_1_ is the cytochrome binding site.

**Complex 3** (Figure 6) has many names: coenzyme Q, cytochrome *c* oxidoreductase, and cytochrome *bc_1_* complex. It adds electrons to cytochrome c and moves two protons across the membrane while releasing two other protons from ubiquinol as discussed in [123].

**Figure 7:**
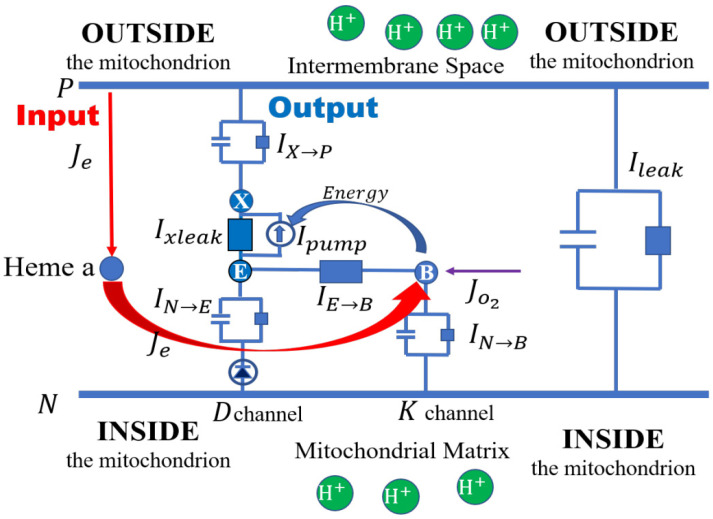
**Complex 4**. A reasonably oversimplified model of cytochrome c oxidase and associated current flows: symbols defined and explained in text and Reference [12]. The meaning of each term in the figure (e.g., D channel, K channel, I_pump_) is complex and is specified in detail in Reference [12].

**Complex 4** (Figure 7) also known as cytochrome c oxidase is a protein complex containing many subunits. The complex contains two hemes, a cytochrome a and cytochrome a3, and two copper centers, the CuA and CuB centers. It is a cathedral of atomic design of great biological importance and so has been studied extensively [124,125] for a long time [126]. It has been modeled with powerful techniques in the chemical tradition using master equations [118,119] and with variational methods of the theory of complex fluids [1] (pioneered by Chun Liu [97,127]) applied to complex biological structures [128]. The approach advocated here and attempted in [1] buttresses the master equations of [118,119] with the universal laws of current flow.

Cytochrome c oxidase is the last enzyme complex in the electron transport chain which delivers protons to ATP synthase; see Figure 2. Cytochrome c oxidase is where the electron transport chain delivers electrons to oxygen (from cytochrome c), yielding two molecules of water (H_2_O). The complex transfers four protons across the membrane. Both electron and proton flows are currents that follow Maxwell equations and current law, e.g., the HHK Equation (1). Figure 7 is drawn without conformational changes because “cytochrome c oxidase … seems to work almost purely by Coulombic principles without the need for significant protein conformational changes” [125].

The circuit in Figure 7 is drawn to show the minimal circuit complexities needed to deal with the main function of cytochrome c oxidase, namely the generation of proton flow. Important chemical details are found elsewhere in the literature [125,129,130]. The experimental literature provides experimental details that exceed what is needed to describe electrical properties and current flows. Of course, one of the advantages of the circuit approach adopted in this paper is that it rarely needs atomic detail. Atomic detail is not often needed in descriptions of electronic circuits of our computers. Atomic detail was not used by Hodgkin and Huxley [23,66] to compute the propagating action potential of nerve fibers. However, some atomic detail is needed on occasion. ***Figure 7 will surely need additional detail to deal with the wealth of experimental data available***.

A circuit analysis of Figure 7 has been completed [1] which includes a description of all of the chemical reactions shown in the figure. The constraints of the HKK Equation (1) are built into the model itself and include the role of the membrane capacitance in the circuit shown. ***The analysis allows the prediction of any current for any input under any conditions of interest***. More than forty graphs are shown in [1] to show the utility of this approach.

A serious limitation of this kind of model is its lack of detail in describing the switch that prevents backflow [111,112]. This issue is central to all models of active transport [111,112,119,129,130,131], including our own: conformational change does not, in my view, explain the mechanism because it is not based on ***physical*** properties of the protein and its structure. The description “conformational change” does not permit quantitative predictions in a range of experimental conditions, in contrast to the predictions in a wide range of conditions made by the rest of the electro-osmotic model [1]. In my view, theories of protein structure need to be extended to deal with protein conformation in a physically consistent manner: molecular dynamics must be extended to deal with the electric field more realistically to solve this problem. Molecular dynamics today uses periodic boundary conditions, and these seriously distort long-range electric fields and flows. Work has started in that direction [132,133,134] although it has not included Maxwell current laws, as far as I know. Maxwell current laws have been used to analyze the near switching (“gating”) behavior of the voltage sensor of sodium channels [135]. Gates that activate and inactivate can provide the switching needed in transporters [136,137,138].

It is important to realize that the switch involved in all of these transporters is in essence an extreme form of rectification. Rectification occurs in any fixed-charge system when the charge changes sign. This is the mechanism of **PN** diodes that underly the behavior of transistors. The shape of the electric field changes when the diode is forward-biased or reverse-biased. The rectification allows current to flow or not because it includes a large potential barrier or not. These barriers and this rectification do not depend on any significant change in the spatial distribution of mass, i.e., it does not depend on conformational change in the normal sense of the word as used in protein chemistry. Rather, it depends on the change in the spatial distribution of the electric field, i.e., it depends on the conformation (i.e., the shape) of the electric field, without violating the dictionary meaning of the word “conformation”. This issue is discussed at length in [1] which includes an historical perspective as well. Switching of this sort has been seen in ionic systems, using a biological protein as a template [139] and is now found in nanotechnology [140] and even in practical technological applications [140,141,142].

## 3. Discussion

**Electron transport is an electrical current**, as is proton transport and the transport of metabolites like NADH and FAD. In particular, electron transport in a quinone chain remains a current, rather like electron transport in the delocalized electron orbitals of wires of our technology.

Electric current has properties of its own that make analysis of charge transport quantitative and at the same time much easier than in classical chemiosmotic theory. The paths for electrons form circuits for currents and can be analyzed that way, just as paths for electrons in wires are analyzed by classical methods of circuit theory.

The chemiosmotic theory brought electricity into view as an essential part of respiratory metabolism [8,9,10,12,111,112,113,120]. In that theory, proton **motive** force and the electron **transport** chain emerged as central players in ATP synthesis. Together, they provide the proton **flow** that powers ATP synthase as it generates ATP (Figure 2). The bold-faced words of the last sentences show the importance of movement in respiratory metabolism and the chemiosmotic hypothesis itself.

The figures of this paper were chosen from the existing experimental literature to show flow inside each component of the respiratory chain in detail. The flows culminate in the proton flows (shown in Figure 2B) that sum to drive ATP production by ATP synthase. The figures are taken from public domain sources to emphasize the widespread acknowledgement of the importance of flow. The cited videos are even more eloquent in that regard. What seems unmentioned, however, is that these flows are electrical currents. What seems unmentioned is the knowledge of physical scientists concerning the flow of current and the laws of electrodynamics that govern those flows, whether between stars or in mitochondria. The flow of current requires numbers and equations for analysis. The chemiosmotic theory is qualitative in essence and does not depend on the known physical properties of electricity or current flow or the equations that describe them. The electro-osmotic approach includes the proton movements and the proton motive force that helps to drive them just as it includes all ion movements (and capacitance and displacement currents) and the driving forces that cause them.

**History:** Movement seems to have been overlooked in the ***analysis*** of the respiratory chain in contrast to its role in visualizations of models. Movement is shown vividly in widely available figures, as reproduced here, and videos emphasize it. But the movement seems not to have been analyzed. A chemiosmotic theory that does not include current has been used when an electro-osmotic theory including current was needed to analyze the models with their visualizations of transport.

Electro-osmotic theory is needed because moving charge creates an electrical current subject to its own rules beyond that of the conservation of matter. The total current across the total mitochondrial membrane must sum to zero at any time, however short, according to the HHK Equation (1) and the Maxwell equations. Integrated over long time scales, the fluxes of chemical species also add to zero. But ***the fluxes of chemical species do NOT have to sum to zero on a short time scale***, e.g., that of ATP synthase, as we have discussed.

Electrons were recognized as the current carriers of classical electrodynamics, in wires, for example, ever since electrons were discovered [143,144]. Electrons in the electron transport chain play the same role. (The role of true (total) current was well understood by Maxwell, long before the discovery of the electron, interestingly enough [41,42]). The special properties of current are described in equation **A** of the Maxwell equations [40], p. 465. The Kirchhoff laws of current flow were known before that. They were used extensively, for example, in the design and use of telegraphs almost two hundred years ago. The current laws, however, receive little emphasis in many texts of electrodynamics today. The Kirchhoff current law is not included in the index of the broadly cited advanced text [145] or the widely used introduction [3]. Zangwill’s modern advanced text [4] presents Kirchhoff’s current law on p. 524 in a form that contradicts the continuity equation presented on p. 32.

It is unclear why the rules of current flow have been neglected in texts of electrodynamics. Perhaps the inability to derive current laws from the properties of individual charges was embarrassing to textbook authors from Abraham going forward [146,147]. That neglect makes it easier to understand why the rules of current flow have also been neglected in the study of the electron transport chain of mitochondria. Those rules may also have been neglected because they are inherently quantitative and mathematical and require a different training and approach from that used in qualitative biochemistry, for the most part.

**The Maxwell Equations are Difficult Constitutive Equations:** The classic form of the Maxwell equations involving the D field are hard to understand. Experimental data shows that the use of a single dielectric constant in the classical Maxwell equations cannot deal with the movements of charge in the ionic solutions of life in the references cited previously [14,148]. See Appendix A and Appendix B for further discussion and documentation.

The relevance of the ***classical*** Maxwell equations to biological systems is obscure because they involve an over-approximated dielectric approximation. The classic Maxwell equations are then easy to ignore. This seems to have been the case in the chemiosmotic theory. The dielectric approximation used in the definition of D is obviously unable to deal with the various forms of charge movement in proteins. The hierarchies of structures that make up proteins involve an enormous range of motions from angstroms to microns. The resulting charge movements cover a vast range of times, from femtoseconds to seconds, to minutes. A single dielectric constant obviously cannot describe such variations in charge density when proteins respond to the electric field and polarize [14,148]. In fact, the idea of polarization itself has been severely criticized as ill posed in a text by a Nobel Laureate, see Appendix A and Appendix B for relevant Maxwell equations and a rigorous mathematical proof of the ill-posed nature of the usual idea of polarization.

The classical form of the Maxwell equations cannot deal with this range of phenomena (with a single dielectric constant) because the classical Maxwell equations are in fact constitutive equations that depend on constituents [149]. Constituents vary, so the classical form of the Maxwell equations varies. They are not universal.

The dielectric approximation is nonetheless an important teaching tool and a necessary approximation for models investigating new phenomena or seeking a low-resolution understanding. It is widely and appropriately used for that reason. But the dielectric approximation must be used with care, and treated as the over-approximation that it actually is.

**Electrodynamics as a universal theory of “everything electrical”** emerges when the dielectric approximation is replaced by the core Maxwell equations and an explicit model of polarization [19]. When core Maxwell equations are written, they do not include material constants and are universal in that sense. What is not apparent or at least was not to me for a long time, is that core Maxwell equations could be useful as well as universal.

Why should equations that do not specify specific properties of polarization and dielectrics be of any general use, when everything that the equations describe have specific (and often diverse) dielectric properties? The answer to this question lies in the current laws implied by the core Maxwell equations.

The current equations are both universal and useful because they are corollaries of the Maxwell equations that do not depend on the properties of matter. They only depend on the electrical and magnetic constants or the speed of light. Because they are universal and as exact as any known physics, the current laws serve as the basis of our electrical and electronic technology.

The current laws are derived by applying the divergence operator to both sides of the Maxwell Ampere law. Then, a universal current law appears that is useful everywhere and does not depend on dielectric properties or polarization in any way. The key property then depends only on mathematics: the divergence of the curl on the left-hand side of the Maxwell Ampere law is always zero by a general result of vector calculus well known to physicists and mathematicians. The divergence of the right-hand side (which defines the total current) is also zero. The total current does not accumulate because it has zero divergence. Maxwell clearly understood that his total “true” current does not accumulate, ever, anywhere, at any time, according to his field equations [38,40,41].

**Current laws** derived from the Maxwell equations are helpful as well as universal. The Kirchhoff current law (and its generalizations) has been used innumerable times to design the electrical and electronic technologies of human technology, since more or less 1850, and of course, in the digital technology we rely on today. In a very practical sense, Kirchhoff’s current laws (Appendix A and Appendix B) are the most used application of electricity, because billions of people use computers every day and each computer contains billions of circuits designed with those laws. It is all the more surprising that the word Kirchhoff does not appear in the beautifully sculpted introductory electrodynamics text by Griffiths [3] or the widely used treatise of Jackson [145].

It is clear that all theories of ion movement must satisfy the core Maxwell equations and the current laws that are corollaries of the core Maxwell equations. ***Modern science does not allow vitalist exceptions, even when complex biological structures perform vital functions***, like generating ATP in mitochondria and chloroplasts. In fact, this paper shows that universal current laws simplify the analysis of such complex biological structures, much as they simplify the analysis of complex engineering structures in our digital technology [52].

Current laws do not fully describe systems like the electron transport chain of mitochondria until they are combined with the rate constant laws showing how substrates and chemical reactions combine with each other. No doubt, the classical laws of chemical reactions oversimplify the dependence of their rate constants on electrical potentials [84]. This issue remains to be dealt with in future work [150].

Current laws have been combined with classical descriptions of chemical reactions to compute the properties of cytochrome c oxidase [1]. Diffusion, convection, and electrical migration were combined using the theory of complex solutions and its variational approach. Complex biological structures and systems have been studied in this way [1,128,151,152,153,154,155,156,157,158,159]. One can hope that similar methods will be useful in understanding the respiratory complex in general, as it uses electron transport to create proton flows in the protein complexes of mitochondria, as the proton flows create ATP in the exquisite machine of ATP synthase.

## 4. Conclusions

Current laws are needed to analyze the flow of electrons and protons, as they generate ATP in mitochondria and chloroplasts. Chemiosmotic theory must be replaced by an electro-osmotic theory of ATP production that conforms to the Maxwell Ampere equation of electrodynamics while still including proton movement and the proton motive force. The proton motive force is included in the electro-osmotic approach as just one of the components of the total electrochemical potential. Circuit analysis includes its role just as it includes the role of any other ionic current.

## Data Availability

No new data were created or analyzed in this study.

## Appendix A

**Polarization in the Maxwell Equations:** The Maxwell equations as ordinarily presented are constitutive equations that depend on the properties of materials in a particular approximation. They represent the polarization response of material to a change in electric field in a dielectric approximation that is severely criticized by a Nobel Laureate [160], who says on p. 507, “This example teaches us that in the real atomic world the distinction between bound charge and free charge is more or less arbitrary, and so, therefore, is the concept of polarization density P.”

The difficulty with polarization can be seen by purely mathematical arguments as discussed at length. An extract—lightly edited—is included here so this paper is complete in itself: “The ambiguity in P in the Maxwell differential equations means that any model $P_{model}\left(x,y,z|t\right)$ of polarization can have $curl\;\tilde{C}\left(x,y,z|t\right)$ added to it, without making any change in the $\mathrm{div}P\left(x,y,z|t\right)$ in the Maxwell version of Gauss’ law. In other words, the polarization $\mathrm{div}P\left(x,y,z|t\right)$ does not provide a unique structural model of polarization $P_{model}\left(x,y,z|t\right)$. In particular a model drawn from an atomic detail structure can be modified by adding a polarization $\tilde{P}\left(x,y,z|t\right)≜curl\;\tilde{C}\left(x,y,z|t\right)$ to its representation (i.e., “drawing”) of polarization without changing electrical properties at all: $\mathrm{div}P\equiv \mathrm{div}\left(P+\tilde{P}\right)$.”

Models of the polarization $P_{model}^{1}$ and $P_{model}^{2}$ of the same structure written by different authors may be strikingly different but they can give the same electrical forces and fields. Misunderstanding and unproductive argument result: “What is the true description of a dielectric object (e.g., protein)?” is a question likely to arise and be unanswerable if the polarization field P is itself not unique. If the field cannot be defined mathematically, polarization is unlikely to be productively definable by words.

Obviously, if polarization P is an arbitrary idea, so are equations like the classical Maxwell equations that embody P. The core Maxwell equations do not suffer from this defect.

## Appendix B. Derivation of Kirchhoff’s Law

The suggestion was made that this paper should include material previously published so it would be more self-contained. The following is a derivation of the current laws, both Kirchhoff (see Appendix B) and Maxwell current laws, quoted from Reference [7] with permission.

“We start with the Maxwell Ampere law which is one of the Maxwell equations that describe electrodynamics without significant error [4,5]. And we define total current as Maxwell did.

(A1)
$$\mathrm{curl}\;B=\mu_{0}(J+\varepsilon_{0}\partial E/\partial t)=\mu_{0}J_{\mathrm{total}}\;\;\;\;\;J_{\mathrm{total}}=J+\varepsilon_{0}\partial E/\partial t$$

Take the divergence of both sides using an identity of vector calculus [20,161,162] that is part of the general Helmholtz decomposition of vector fields [5,161]. Quaitative discussion of the “div curl is zero” theorem is in the main text.:

(A2) $$\mathrm{div}\;\mathrm{curl}\;B=0=\mathrm{div}\left(\mu_{0}(J+\varepsilon_{0}\partial E/\partial t)\right)=\mathrm{div}(\mu_{0}J_{\mathrm{total}})$$

This establishes the three-dimensional version of a current law:

(A3) $$\mathrm{Maxwell}\;\mathrm{Current}\;\mathrm{Law}\;\mathrm{in}\;\mathrm{Three}\;\mathrm{Dimensions}:\;\mathrm{div}J_{\mathrm{total}}=0$$

We are motivated to use the variable $J_{\mathrm{total}}$ = $J+\varepsilon_{0}\partial E/\partial t$ because Maxwell gave it special significance as “one of the chief peculiarities of this treatise” [41]. He called it the true current. He could hardly have chosen a stronger adjective than “true”. Maxwell was explicit about why he used the name true current. He said that “ … estimating the total movement of electricity [requires] an equation of true currents” like the Maxwell Ampere law Equation (A1). See Volume 2, Section 610, p. 232 of his “A Treatise on Electricity and Magnetism” [42].

The treatment here, of course, depends on mathematics not Maxwell’s opinion of what was true.”
